# Demurrage and detention: from operational challenges towards solutions

**DOI:** 10.1186/s41072-023-00132-1

**Published:** 2023-03-20

**Authors:** Katrien Storms, Christa Sys, Thierry Vanelslander, Ruben Van Deuren

**Affiliations:** Department of Transport and Regional Economics, University of Antwerp, Antwerp, Belgium

**Keywords:** Demurrage, Detention, Container shipping, Free time, Intermodal transport, Maritime supply chain

## Abstract

Reduced free time and increased fees for demurrage and detention create organizational challenges with respect to intermodal transport. As a result, actors within the maritime supply chains are confronted with greater complexity and higher risk of costs; and, therefore, often fall back on transportation by truck to and from the hinterland. That is why the present research examines the impact of this evolution on the bottom line of the involved actors from a maritime supply chain perspective. The research approach consists of reviewing the relevant literature, analyzing the available sector data obtained through interviews and professional experience, and validating the proposed solutions. Starting from the research results, the problem-solving discussions resulted in the following top three as feasible solutions: digitalization, extra ‘free time’ for hinterland locations, and more attention during the negotiation process.

## Introduction

Since the White paper in 2011, the European Commission set a goal to develop a competitive, intermodal and sustainable freight transport system by, amongst other things, shifting inland transportation from road to inland waterways and rail transport. However, over the years, it became clear that this goal is particularly difficult to achieve as in 2019, road transport still takes up 76.3% (based on tonne-kilometers) of the inland freight transport in the European Union (EU) (Eurostat 2021). The European Green Deal (2019), the Sustainable and Smart Mobility Strategy (2020), NAIADES III Action plan 2021–2027 (2021a), and Fit for 55 (2021b) reaffirm the need and importance of this modal shift to evolve towards a more viable transportation system.

While in practice, the reduced free time^1^ and increased compensation fees for demurrage and detention (D&D) set by carriers create organizational challenges with respect to intermodal transport. Under the terms 'demurrage and detention' is understood "the compensation paid by the shipper or receiver to the shipping company for the delay of the container at the terminal (demurrage), and/or in the chain (detention) if the agreed period (free time) has expired" (Storm 2011; Federal Maritime Commission 2015; Chaudhri 2016). Uncertainties in practical operations with respect to intermodal transport make that actors within the maritime supply chains are confronted with greater complexity and higher risk of costs, both of which have severe consequences on the shipper’s bottom line (Manaadiar 2019); and therefore, shippers (including freight forwarders and others acting on their behalf) often fall back on road transport, hence ‘reverse’ modal shift. The above-mentioned developments result from changed practices of shipping companies at the headquarters level. This is, of course, completely against the social trend towards more sustainable transport.

The academic literature review shows that only limited research on D&D is available. Storm (2011) indicated that little attention is paid to D&D because the related costs are relatively low compared to the total transportation costs. However, D&D is an issue that gets mentioned and appears in sector-relevant publications more frequently (Federal Maritime Commission 2015; FIATA 2018; Federal Register 2020). Moreover, the sector analysis made it quickly clear that the topic is high on the agenda. Since 2015, the shippers’ agitation over increased D&D fees has been intensifying due to recurring US port disruptions, the bankruptcy of Hanjin, the cyberattacks on the information technology and communication systems of Maersk Line and other carriers, and, more recently due to COVID-19 (labor shortage, port congestion, container availability) and the blockage of the Suez Canal.

The present paper aims to clarify commonly used terminology, describe factors that contribute to D&D, identify the cost elements of D&D, and validate feasible solutions. Therefore, the research question arises whether D&D functions properly using a supply chain approach.

This study contributes to the literature in four ways. Firstly, the paper gives a comprehensive overview of all relevant terminology including operational issues like a pass-through of the charges on other actors. So, the second major contribution is related to the fact that D&D have undeniably impacted various stages throughout the maritime supply chain (Storm 2011). Thirdly, the industrial-economic approach in close cooperation with the industry is a novel element in the research. Lastly, the research provides an insight into the cost elements of the shipping companies linked to the management of their container inventory (a.o. due to the cost charged by the terminal for the storage of containers, due to the monitoring of (reefer/dangerous goods) containers). In a free-market economy, this indicates a capacity problem solved via the price mechanism (Lipczynski et al. 2017).

The structure of the remainder of the paper is as follows. Section "Research approach" elaborates on the three building blocks of the research. The "Literature review" Sect. summarizes academic and sector-relevant literature. Furthermore, it describes the factors that contribute to the risk of attracting D&D. Section "Results" defines the research population and the solution-oriented approach and explains the research findings. The paper ends with the "Conclusions, recommendations, and further research" Sect.

## Research approach

**Fig. 1:**
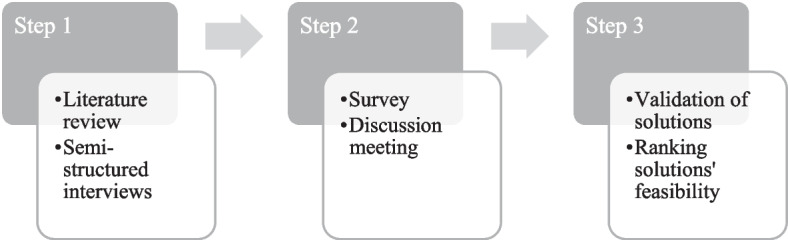
Research approach. *Source*: own composition

Research to date has not yet identified validated feasible solutions for the D&D problem. Therefore, this paper opts for a three-step approach (Fig. 1).

Step 1 focuses on the literature on D&D from the container shipping industry's point of view. Hence, a review of the literature and sector-relevant publications over the last two decades is given. The literature review findings, combined with information retrieved from semi-structured interviews by Chaudhri (2016), are input for step 2 of the research approach.

Step 2 considers that container D&D is a process that involves multiple stages of the supply chain. Therefore, it is essential to take different perspectives and willingness towards initiatives into account to increase the chance of success of a proposed solution. With this in mind, a problem-solving discussion meeting following the Chatham rules was organized on September 5th, 2017, on "Demurrage and Detention, or more specifically, whether or not it functions properly.” The 53 attendees active in the maritime ecosystem of the port of Antwerp,^2^ Belgium, represented different stakeholders of the maritime supply chain confronted with the identified issues. As the 2nd largest port in Europe, the port of Antwerp and its stakeholders are a good representation of D&D practices which also manifest in other ports. Moreover, the questioned stakeholders are most often global operators, active in various continents. The purpose of the discussion meeting was to share knowledge (e.g., terminology, own experience), to discuss solutions (e.g., promote intermodality), and to network (e.g., get to know other actors also confronted with this problem). Given the commercial sensitivity of the D&D matter, the participants were asked to complete a survey before the discussion meeting.

In step 3, the sector validates the solutions. This was done by asking the sector to rank the solution in terms of feasibility, arguments for/against the solution, and who should take the initiative. In addition, during the COVID-19 pandemic, individual online meetings took place with high-level management of shipping agents, terminal operators, logistics service providers, and shippers, who are confronted with this problem almost daily (S. Declercq, personal communication, April 13, 2021; I. Verdonck, personal communication, April 13, 2021).

## Literature review

The research started with a literature review covering the 2000–2021 period. Academic literature covering the subject of container D&D is limited. On the other hand, the issues linked to D&D regularly appear on the discussion table of regulatory bodies (Federal Maritime Commission) and sector associations such as the International Federation of Freight Forwarders (FIATA), European Association for Forwarding, Transport, Logistics, and Customs Services (CLECAT), and European Shippers Council (ESC). Their views are extensively discussed in sector-relevant publications, which are also included in the present study.

In this section, first, the terms D&D will be explained. Then, the issues related to D&D are discussed. Finally, the organizational practices and challenges of terminals and shipping lines are set out.

### Definition of demurrage and detention

Gaining an understanding of the terminology using search portals like Scopus, Web of Science, and Google Scholar quickly made it clear that the terms are used loosely. An explanation can be found in the fact that the term 'demurrage' is used as a fair compensation for the use of capacity, be it ship, container, etc. The general idea behind this charge is an incentive to return equipment (ship, container,…) as soon as possible and not an unexpected additional freight cost.

The concept of ‘demurrage and despatch’, used in bulk cargo shipments, has existed for more than 100 years. The first principles of demurrage claims in the maritime sector were established at the beginning of the nineteenth century. The Merchant Shipping act of 1876 described the first official definition of detention as withholding accidentally or by design a person or a thing (Rapalje & Lawrence 1997; Storm 2011). The ‘Demurrage and Despatch’ rules in the Voyage charter party lay-time rules 1993 (Schofield 2016) more specifically stated that when a vessel is delayed beyond laytime for which the owner is not responsible, an agreed amount has to be paid to the owner of the vessel. While despatch could be described as an incentive clause in the charter party (C/P) if the handling of the ship is completed in a shorter period as agreed. Demurrage and despatch are thus a longstanding practice for chartering vessels. In short, the principles refer to the concept of ‘late return of equipment’ (capacity).

Due to the growth of containerization and the associated capacity challenges (equipment, space), in parallel to ‘Demurrage and Despatch’ (C/P), the terms ‘Demurrage and Detention’ related to the use of containers in the contract between carrier and shipper started to develop (Schofield 2016). The term ‘demurrage’ refers to the charge that arises inside the terminal when the container is not retrieved within the free time. Since the maritime terminal operator (MTO) has no contract with the shipper, it will charge the shipping line storage costs if the loaded export/import container is positioned at the terminal for a longer period than the contracted free time. The shipping line will pass on these costs to the shipper or actors acting on behalf of the shipper by incorporating them in the export/import demurrage charge (Storm 2011; Yu et al. 2015; Jeong et al. 2020). The term ‘detention’ is similar to a demurrage charge but arises outside the terminal. Similarly, the shipping line will charge the receiver a detention fee if the container is not returned within the agreed detention threshold interval. The present research focuses on fair compensation for the usage of containers in Europe. Hence, the literature search used both ‘demurrage’ and ‘detention’ as keywords. Inconsistent use of the terms ‘demurrage’ and ‘detention’ are found in the academic literature and sector-relevant publications, incl. websites of container carriers (Chaudhri 2016; Storm 2011; Federal Maritime Commission, 2018a, 2018b). Essentially, D&D is a charging structure, only active when a carrier’s container is not collected and/or delivered within the allowed free time.

Kim and Kim (2007) define free time as the period during which a container can be stored and used without any charge. Chaudhri (2016) gives a more detailed definition of free time: “the duration of days, calendar or working days, which are pre-negotiated or determined between the carrier and the shipper, before the imposition of any D&D charges.” When the shipper cannot pick up and drop off the loaded container within the free detention period, the shipping line will charge the shipper a daily fee (Subramanian & Kuvar, 2018).

**Fig. 2:**
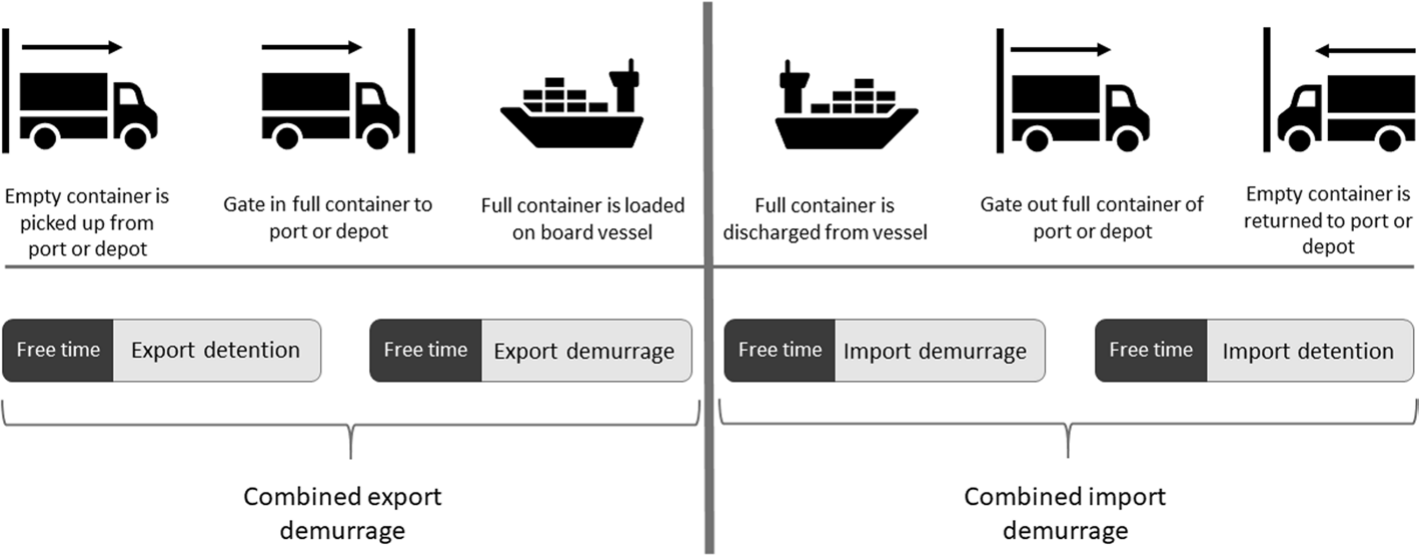
Structure of demurrage and detention. *Source*: own composition based on Storm (2011) and Maersk (2017, 2021)

Figure 2 shows the structure of D&D in more detail. Since a container does not have the same user along the supply chain, the D&D process must be divided into two parts: export/import demurrage and export/import detention (Storm 2011; Fazi & Roodbergen 2018).

Furthermore, the term 'combined (export/import) demurrage and detention' takes the demurrage and detention period together, resulting in a single free period (Storm 2011; Fazi & Roodbergen 2018). An example illustrates: the most common and well-known form is if, for example, a shipper gets seven days of free time at the terminal and an allotted free time of seven days for the usage of a container outside the port. If combined D&D applies, the shipper has a period of 14 days in total. The shipper, then, can choose how many days are used for demurrage and detention (e.g., five days free time demurrage, nine days free time detention, or ten days free time demurrage, four days free time detention, etc.).

### Issues related to demurrage and detention

The literature review further indicates that there are still some other issues linked to D&D, namely international commercial terms and the carriage of the container (merchant versus carrier haulage).

First, it becomes clear that various parties (shipping lines, terminals, shippers, and their service providers (freight forwarders, intermodal companies, etc.) are involved in the D&D matter (Storm 2011; Chaudhri 2016). Therefore, tackling the problem will be difficult as there are not always mutual contracts with one another. To start, for example, via the International Commercial Terms (also known as the Incoterms, currently Incoterms 2020®) in the sales contract between the seller and the buyer of goods, the commitments, costs, and liabilities of the two actors in the international trade are arranged. In other words, choosing a particular Incoterm determines the seller’s and buyer’s level of costs, risks, and organization of transporting the goods. In the case mentioned above, the seller and buyer can have chosen Incoterm Cost and Freight (CFR), which means that the seller is liable for the export D&D, and the buyer is responsible for the import D&D. However, when the seller and buyer set Incoterm EX Works (EXW), the buyer will have to arrange the entire transport from the seller’s premises to their own premises. Consequently, the buyer will be responsible for both export and import D&D.

Furthermore, depending on the chosen Incoterm2020®, either the seller or the buyer must arrange the contract of carriage and the Bill of Lading with the shipping line. The choice of Incoterm2020® in the sales contract between seller and buyer thus influences which actor is in a contract of carriage with the shipping line. Nevertheless, the Merchant Clause in the Bill of Lading makes it possible for the shipping line to impose the D&D charges on any other party included in the carriage of the goods, such as the freight forwarder and trucking company (Magrath 2020; Munday 2021). In addition to the relation shipping line—seller/buyer, the shipping line has an agreement with the terminal operator to handle and store the shipping line’s containers. When the stored containers are overdue, the terminal operator will issue storage charges to the shipping line. The shipping line will pass on the terminal operator's storage costs to the shipper or receiver by including these costs in the demurrage charge (Storm 2011; Federal Maritime Commission 2015). Therefore, the number of free days a shipper gets for the D&D period will be equal to or less than the terminal dwell time. The shipper or receiver has only an operational relationship with the terminal operator and no contractual one (Storm 2011; Federal Maritime Commission, 2018b). This makes the entire process of D&D very complex as the actor responsible for the demurrage and/or detention charges is often not the bearer of these charges (Chaudhri 2016; Federal Maritime Commission, 2018a). Hence, multiple elements are at play to determine the D&D free period, the actor charged, and the D&D tariff level, i.e., incoterms, the Bill of lading, and terms of carriage (Federal Maritime Commission 2015; Magrath 2020).

Third, shipping lines and terminal operators are prominent players who, in recent years, have tried to gain more control over hinterland transport operations (Meersman et al. 2010; de Langen et al. 2013; Gubbi et al. 2014; Yu et al. 2018). An example is the shipper’s choice to transport a container either under carrier haulage or merchant haulage. When a shipper decides to transport the container under **carrier haulage**, the shipping line organizes its entire transport until its final destination. This gives the shipping line more control over the container for a more extended period as the receiver is only responsible for the unloading of the container. When a container is transported under **merchant haulage**, the shipper has to select a local carrier for inland transport as the shipping line is only responsible for the sea transport. In other words, the shipping line loses control over the container for the entire inland transport part as well as the unloading. The receiver, however, has more flexibility and more contractual power to negotiate inland transport rates (Fazi & Roodbergen 2018; Yu et al. 2018). Shipping lines use the type of carriage to differentiate between charging the D&D fee or not. FIATA (2018) reports that shipping lines might abuse their position by charging D&D fees for merchants who arranged transport under merchant haulage but waiving D&D fees for merchants who organize their transport under carrier haulage.

### Organizational practices and challenges of terminals and shipping lines

In recent years, the practice of D&D set by carriers has led to repeated discussions between shippers and carriers. The issue of D&D is first explained in light of some recent events. Then, the capacity of maritime terminal operators and carriers is shortly discussed. Furthermore, the organizational challenges of terminals are laid out. Finally, several organizational challenges of shipping lines are clarified.

#### The issue is not new

The changes in D&D are not just a local phenomenon but a global trend. Moreover, the issue of D&D is not new (Mongelluzzo 2000, 2005, 2020; Leach 2005; Federal Maritime Commission 2015, 2018a, 2018b; Federal Register 2020).

In 2000, the ports of Los Angeles and Long Beach on the West Coast asked their harbour commissions to reduce the amount of free time for both import and export containers to reduce congestion at the terminals (Mongelluzzo 2000). Leach (2005) stated that the increases in D&D fees and the cutting of the free-time period started discreetly in the US, on the East Coast by Maher Terminals. Rapidly, the other terminals in the Port of New York followed, and also the rest of the world gradually adopted these trends. In 2015, the Federal Maritime Commission (FMC) published a report regarding the increasing concerns of importers, exporters, and transportation companies related to D&D charges by MTOs and vessel-operating common carriers (VOCCs) in the main US gateway ports for terminal delays over which they had no control. A problem which shippers are regularly confronted with is MTOs telling them that it is impossible to pick up the container due to gate delays and on-dock congestion. Additionally, when the terminal permits the shipper to get the container, the shipping line or MTO will not release it until the demurrage fee is paid. Besides the inability of a shipper to pick up the container at the terminal, there are also issues with the returns of empty containers as MTOs and VOCCs put a limit on the days and shifts or even restrict the returns of empties. Hence, importers, exporters, and their agents wonder who should be held responsible for these delays. Consequently, they believe that terminal operators’ and shipping lines’ D&D practices are unfair (Federal Maritime Commission 2015).

The issue of D&D came higher on the agenda due to these recurrent US port disruptions, the bankruptcy of Hanjin, and a series of cyber-attacks on shipping lines. In February 2017, the Seoul Court declared the South Korean shipping line Hanjin bankrupt. Hundreds of thousands of containers were delayed making the bankruptcy of the once seventh-largest container carrier one of the biggest in the container shipping industry since the bankruptcy of US lines in 1986. The US bankruptcy court decided that Hanjin will not levy demurrage charges for late containers (Dupin 2016; Hand 2017). Also, in 2017, Maersk had to waive the D&D charges, as the containers could not be released due to the cyber-attack, which completely shut down the information technology and communication systems (Knowler 2017). In 2018, the Federal Maritime Commission held, due to the rising number of complaints, a two-day hearing to discuss the problems regarding D&D, with which shippers, freight forwarders, shipping lines, truckers, and terminal operators are confronted. Two years later, in 2020, the Federal Register assessed the fairness of D&D and published the following: “Failure of carriers and MTOs to operate in a way consistent with the Interpretive Rule on Detention and Demurrage that became effective on May 18th, 2020, might constitute a violation of 46 US Code § 41102 (c) which prohibits unjust and unreasonable practices and regulations related to, or connected with, receiving, handling, storing, or delivering property” (Federal Register 2020). The Interpretive Rule and its Incentive Principle are also highlighted in the FMC’s Fact Finding Investigation 29. However, due to COVID-19, the question arises whether this Interpretive Rule guideline is sufficient as the congestion problems at marine terminals, shortages of skilled labor, and available chassis in the US are skyrocketing, making the D&D charges uncontrollable. Therefore, a coalition of truckers, shippers, and customs brokers asked the FMC to turn the guidelines into rules that suspend the D&D charges (Mongelluzzo 2020). On June 16th, 2022, the US Congress incorporated the Interpretive Rule in the new Ocean Shipping Reform Act of 2022^3^ (OSRA22) (Senate—Commerce, Science, and Transportation, 2022). OSRA22 gives the FMC the authority to administer sanctions for violations linked to OSRA22. The new law focuses on the American market. Since the latter is known for higher D&D charges and is organized differently (e.g. using chassis) than the European market, the present study concentrates on the European market. Also within Europe, shippers and forwarding agents indicate that the practices of carriers regarding container equipment, tariffs, and D&D charges are unreasonable and a risk to the possible economic recovery in Europe (CLECAT 2021; Knowler, 2017).

#### Capacity

The underlying industrial economic theory related to capacity can be used to frame the problem in the relation carrier—terminal and carrier—shipper (Lipczynski et al. 2017). The practice of charging an additional fee for the late return of material is used in various sectors. It is, for instance, a common practice for car rental companies and libraries. If you rent a car or borrow a book and bring it back after the agreed rental period, you will be charged for the late return of the vehicle or book. The charging system called D&D used by shipping lines works similarly as it includes a free period and an overtime fee for the use of the shipping lines' containers. Other players in the maritime transportation system, like sea container terminal operators, also use this practice (Yu et al. 2018). Hence, the objective for shipping lines and terminal operators is the same as their revenue model is based on the number of containers handled. Therefore, they both want to push a container as fast as possible through the container transportation chain (Storm 2011). In the end, it’s all about maximizing the use of assets and thereby maximizing profits.

With respect to the relation carrier–terminal, one of the most important resources of a container terminal is its storage space. The efficient use of this storage space is crucial because it determines the level of productivity and profitability of the container terminal. The container yard at container terminals can be seen as a short-term storage space for inbound and outbound containers. Ideally, the container yard is not used for the long-term storage of containers because it will lead to space that cannot be utilized in a way that will maximize its productivity. High congestion results in lower productivity in handling operations as, in general, more moves have to be made. Terminal operators try to avoid this by setting a pre-specified free period and charging a storage fee to the customer, i.e., the shipping line when the container is stored beyond the free period. The idea behind a short free period and a high storage fee per unit container per day when the dwell time in the terminal exceeds the free time is that it will convince customers to move their containers, while a long free period and a low storage fee will induce the customers to leave their containers at the container terminal (Kim & Kim 2007; Yu et al. 2015). Because of the contractual relationship between the carrier and container terminal operator, the latter charges the former the storage fee for the occupation of limited storage space in the terminal yard (Yu et al. 2018). When carrier haulage is used, the practice has only implications for the carrier. However, when merchant haulage is used, the problem emerges that the terminal operator has no contractual relationship with the shipper or the actor acting on behalf of the shipper, only an operational relationship. The shipping line, then, needs to convince its contracted customers to move their containers.

Therefore, with respect to the carrier–shipper relationship, the shipping line first wants to encourage their customers not to store containers at the terminal for a long time by incorporating the storage charge in the demurrage charge. However, the amount of the demurrage charge that the customer pays to the shipping line is not necessarily equal to the storage charge amount that the shipping line is obligated to pay the terminal operator (Yu et al. 2015). In addition, some shippers try to use as much free time as permitted to save costs (Mongelluzzo 2005; Storm 2011) or are willing to pay the storage charges in order to avoid warehousing costs (Martín et al. 2014). Second, the same principle goes for the use of the shipping line’s containers. A shipping line will charge D&D fees to the shipper and/or receiver to ensure the efficient use of their container equipment. It can be seen as compensation to the shipping line for the use of its material. The shipping line’s goal is to maximize the return on capital investment by increasing the utilization grade of the container. Since the shipping line wants control over the return times, it tries to maintain an uninterrupted container flow by turning them around as quickly as possible. By doing this, it maximizes the number of roundtrips per year. The purpose of D&D is consequently to ensure that a shipper returns the container in time so that the shipping line can reuse it (Storm 2011; de Langen et al. 2013; Fazi & Roodbergen 2018; FIATA 2018; Jeong et al. 2020; Container xChange 2020).

#### Organizational practices of terminals

Over the years, terminal operators had to adapt their organizational practices due to increased ship sizes, terminal congestion, lower labor productivity, and a lack of information. Subsequently, these practices will be described below.

##### Increasing ship size

The size of container ships has grown rapidly over the last years (Sys et al. 2008; Sys 2009; Federal Maritime Commission, 2018a, 2018b; Zweers et al. 2020). In search of economies of scale, the average capacity of a container ship doubled in only one decade. This evolution towards ever-larger container ships affects the ports’ framework and organizational practices of terminal operators to a great extent since the resources and space of container terminal operators are scarce. In addition to the terminal operators’ infrastructural limitation, the shipper or receiver is also affected by the increasing ship size. The shipper or receivers need to know when demurrage starts to run to arrange the container's pick-up. In some cases, shipping lines start counting when the vessel arrives in the port; in others when the container is unloaded from the vessel. In the latter case, the ship’s size affects the start day of demurrage, as it takes several days to unload a vessel fully because of labor shifts, weekends, and holidays. In other words, there is a difference between the day that the first and last container is unloaded from the vessel (Federal Maritime Commission, 2018b; Munday 2021).

##### Terminal congestion

The growth in capacity of mega-ships causes at least three peaks in the cargo handling process at terminals: a peak in ship-to-shore handling, yard operations, and yard-to-hinterland transport handling. First, larger ship sizes mean that call sizes are becoming larger and thus that the cargo volumes that need to be handled are also bigger. This suggests that the number of container crane moves increases. Secondly, due to terminal space issues, MTOs are forced to stack containers higher and tighter, leading to higher occupancy of the yard. Consequently, containers are more difficult to access in congested terminals, and more moves have to be made to retrieve the desired container. This results last but not least in increased peak periods at the landside of terminals which has further repercussions along the hinterland transport chain (OECD/ITF & Merk 2015; Van Hassel et al. 2016; Merk 2018).

To be able to handle the peaks, terminal operators need to adjust their equipment within their current configuration (Sys et al. 2008; OECD/ITF & Merk 2015). The increase in container ship sizes can, for example, be linked to the investment of new (overbridge) cranes allowing high bay storage, the implementation of innovative concepts (e.g., BoxBay by DP World), the need for more automation of the container terminal operations to reach the required productivity levels. This might limit the use of expensive port labour but will probably lead to lower flexibility in peak periods (Merk 2018).

##### Information sharing

Another reason for a container to have a long dwell time at a terminal is the lack of information sharing among the parties involved in the supply chain. As stated earlier, to organize the logistics chain in an efficient way, containers must be delivered and collected on time. To achieve this, all parties involved need information regarding both the arrival (ETA and/or ATA) and departure time (ETS) of the container ships. However, this information is not always known or is not communicated on time to the interested parties. For example, the receiver does not always know the arrival time of an imported container in advance. As a result, the container cannot be picked up in time and will stay at the terminal for a longer period. Imported containers, therefore, often have a longer dwell time at terminals than exported containers, as exported containers are usually delivered on time (Gubbi et al. 2014).

#### Organizational practices carriers

Clearly, a combination of factors led to changed practices by both terminals and shipping lines. Due to the increase in ship size and TEU volumes, terminals had to change their practices by increasing the TEU price per day and limiting free time. Shipping lines responded by changing their practices accordingly by reducing the D&D free time and increasing the charges. However, it seems that the reduced free time and terminal storage charge is not a simple pass-through. The Federal Maritime Commission (2015) states that shipping lines rather than terminal operators generally control the prices, practices, and policies influencing the shippers and receivers directly.

##### Shortening of free time

There is little to no standardization in the number of free days a shipper or receiver gets. Chaudhri (2016) stipulates that the free period has been reducing gradually since the introduction of the container due to capacity issues. In the past, the free period consisted of 30, 21, 14, 10, and 7 days. The typical free period now ranges between 7 and 3 days. Depending on the local practices of the port, the number of available free days can be expressed either in working days or calendar days (i.e., working on Saturdays, Sundays, and holidays). The former is used by most shipping lines to determine the demurrage period. The reason for this is the closure of several terminals on Saturday afternoon and Sunday. The latter is mostly used to set the detention period (Storm 2011; Federal Maritime Commission 2015). This means that the free period and the charges after the free period are calculated on a 7-days-a-week basis, including holidays. Applying working or calendar days can influence the D&D costs to a great extent and has an impact on the decision when to pick up or return the container.

A shipper or receiver has to be able to process the export or import requirements and arrange the drop-off or pick-up of the container to/from the port within this free time window. However, the fast movement of containers is often not possible due to a fault of the shipper or receiver themselves or due to several other drivers over which they have no control, such as customs clearance issues, bad weather conditions, terminal congestion, equipment malfunctions, labour strikes, vessel delays, miscommunication (Container xChange 2020). Extended free time can be granted by the shipping line in certain situations. If not, these events may be used to waive the D&D fees charged during the event (Subramanian & Kuvar, 2018). However, there is no uniformity in whether the shipping line will waive D&D charges due to terminal unproductivity or not. It seems that it depends and varies by customer and situation (Federal Maritime Commission 2015).

##### Starting period of D&D

Besides the lack of standardization in the number of days of the free period, there is also little standardization in the starting and ending point of the D&D period. The exact time when the free period starts or ends may vary between ports and even between terminals at the same port (Federal Maritime Commission 2015). The detention export period starts from the day an empty container is retrieved from the terminal, the gate-out of an empty container, or the reuse of a container and ends with the gate-in of the full container. The day that demurrage export starts is the gate-in of a full container or the day after. The demurrage export period ends when the full container is loaded on the ship or when the vessel starts sailing. There are also several possibilities looking at the starting point of the demurrage import period: the estimated time of arrival of the ship (ETA), the actual time of arrival of the ship (ATA), the day of the release of the container, the day the vessel is completely discharged, the day after the ETA, the day after the ATA, the day after the release of the container, the day after the vessel is completely discharged, etc. According to the Federal Maritime Commission (2018a, 2018b, 2015), import demurrage generally starts on the first day after the container is discharged from the vessel. The import demurrage period ends with a gate-out of the full container. The gate-out of the full container or the day after the gate-out of the full container marks the starting point of the free period of the import detention period. This period ends with the gate-in of the empty container, the day after the gate-in of the empty container, or when the container is reused. The heterogeneity in the starting and ending point of the D&D period can lead to opacity and confusion for shippers, receivers, and their service providers.

##### Higher D&D charges

In addition, the level of the D&D charges did increase considerably during the last years on a global level (FIATA 2018). However, there is no standardization in the tariffs of D&D. The amount of the D&D varies per carrier and is dependent on the tariff per day and the number of days that a container is too late (Storm 2011). The Federal Maritime Commission (2015) investigated the tariffs of six carriers at 32 terminals in the US. The findings show that the average total prices for D&D are higher for importers than exporters and that the prices are higher for demurrage than for detention. When demurrage fees are higher compared to detention fees, it means that the port or terminal is sensitive to congestion (Container xChange 2020). In this case, the shipping line’s primary goal of shortening free time and raising demurrage is to reduce dwell times at the terminal to prevent congestion and thereby improve the overall velocity of the equipment. This, in turn, reduces the shipping line’s equipment inventory needs and its operational costs (Federal Maritime Commission 2015). However, the non-governmental organization FIATA (2018) is concerned that the increased D&D charges and decreased free time defeat its original purpose because it unreasonably increases the transport costs of all actors in the supply chain (FIATA 2018). Mongelluzzo's (2000) and Fazi and Roodbergen's (2018) findings show that D&D fail to reach the intended result. It does not encourage shippers to pick up the containers at the deepsea terminal and, for example, use inland terminals to store them temporarily. In addition, the aforementioned problems and delays at terminals can even result in extra dwell time for the shipping line’s equipment and extra tension among the parties (Federal Maritime Commission 2015). Hence, the lack of uniformity in free time, the starting and ending day of the free time, and the tariffs of D&D among shipping lines make it difficult for shippers, receivers, and their service providers not only to compare rates and practices between shipping lines but also to organize their transport efficiently.

**Fig. 3:**
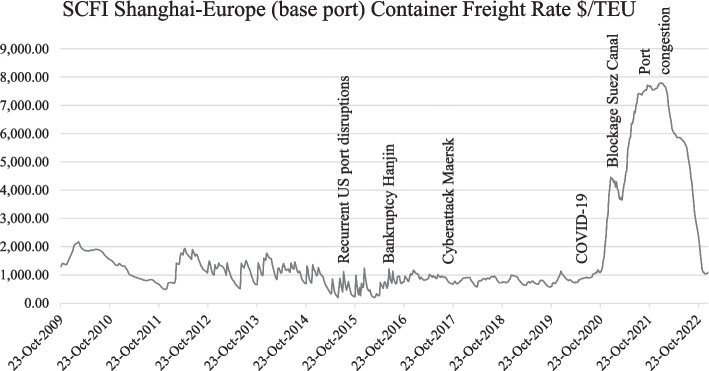
Evolution of Sea freight. *Source*: own composition based on Clarkson Research (2022)

The secondary purpose of D&D is to compensate the shipping line for the use of its containers, as it represents a substantial investment (FIATA 2018; Container xChange 2020). According to Storm (2011), D&D fees are suggested to be an important source of income for shipping lines. Shippers' complaints state that shipping lines do not limit their revenues to their core business, namely the ocean shipment of freight. An increasing number of shippers state that shipping lines use D&D practices not only to gain more control over their equipment but also to generate extra revenue streams. This might indeed be the case as until the beginning of the COVID-19 crisis, the ocean freight prices have been low due to the ongoing sea freight price war between shipping lines. Figure 3 shows the volatility and the downward trend in the freight rates for shipping a container from Asia to Europe. Shipping lines want to compensate for the low freight price by charging higher D&D fees and leaving little to no space for negotiating extra free demurrage or detention time. The D&D practice then functions as a revenue model for shipping lines. When freight rates are higher, D&D is incidental for the shipping line, and the ability to negotiate extra free time is higher (S. Declercq, personal communication, April 13, 2021, personal communication, February 7, 2022; I. Verdonck, personal communication, April 13, 2021). In addition, shippers argue that shipping lines take advantage of the uncertainty in container returns due to port congestion and unforeseen circumstances by imposing higher detention tariffs against the consignee (Storm 2011; Wackett 2019; Jeong et al. 2020).

##### Document closing and physical closing

In the last ten years, the deadline to deliver export containers to the port has been brought forward due to more security measures. The Verified Gross Mass (VGM) and documentation closing have changed from two to five days before the vessel's departure. The physical closing deadline to deliver an export container to the port also increased from two days to three days before the vessel's departure. However, the free demurrage period was shortened to more or less four days. This small time window—earlier closing deadlines and reduced demurrage free time—makes it challenging for shippers to deliver their export containers without incurring demurrage costs (FIATA 2018). In the Best Practices Document, FIATA (2018) further explains the problem by giving the following example: a shipper wants to deliver the VGM or AMS document on the relevant closing day but wants to load the container a few days after the document closing. To complete the VGM closing, the shipper needs the container's number to transport its goods. However, normally the shipping line cannot give the container number until the empty container is picked up. In this situation, the shipper has to pick up the container to complete the necessary documents before VGM closing, load it, and return the laden container before the physical closing. The early delivery of the loaded container to the terminal can, in turn, lead to demurrage charges.

##### Intermodal transport

The number of available free days determines the mode of transport shippers and receivers use to bring the container to the final destination and back. The use of inland waterway transport or rail requires more free days than for trucks and therefore influences D&D. This is an important aspect as the economic and sustainable benefits of using inland waterways and rail will fade if D&D costs take place (Storm 2011). Additional free (detention) days can be granted by the shipping line if the shipper decides to move the container using sustainable modes of transport like barge or train (Chaudhri 2016; Fazi & Roodbergen 2018).

According to Storm (2011), two key drivers for D&D to occur are time and flexibility. The longer it takes to pick up and drop off a container, the higher the D&D costs will be. Time, thus, has a positive effect on D&D costs. Flexibility, on the contrary, influences D&D costs negatively. It determines to which extent shippers and receivers can choose which transport mode to use and when to unload the container within the predetermined free time. Fazi and Roodbergen (2018) revealed that consolidated container transport from the seaport to the hinterland by inland navigation is limited due to the loss of valuable free time and a lack of flexibility in the planning process. When shippers have more flexibility in their modality choice or time of unload, they can react more appropriately to possible delays; in this way, they can minimize the chance of D&D costs. If the granted free period is not sufficient, shippers will choose to transport by truck because it is more flexible and faster (Storm 2011).

##### Negotiations

To mitigate the risk of D&D charges and attempt to offset them, it might be interesting for shippers and/or receivers to discuss the D&D terms when the freight is being negotiated with the shipping line (Chaudhri 2016; Yu et al. 2018). D&D rates are usually unified, making them mostly non-negotiable, but the number of free days can be negotiated. Practically, different consignees can get different D&D free times from a shipping line depending on their relationship, bargaining power, and many other factors (Federal Maritime Commission, 2018a; Yu et al. 2018). For example, shippers with large volumes have the ability and the power to negotiate a longer free period. A shipper can thus try to request additional free days for its cargo which might save him/her D&D costs (Container xChange 2020). If the request is declined, the shipper relies on the standard free days granted by the shipping line (Storm 2011).

##### Reusing containers

Containerization has led to, amongst other things, standardization, low cost, reliable ocean container transport, and ease of handling. However, it also has a disadvantage, namely the empty container movements. Once a container arrives at its final destination, it often has to be repositioned to be able to reuse it, which comes with costs. Legros et al. (2019) investigated the direct reuse of the consignors’ containers in the surrounding area. They focus on the street-turn strategy, which is—according to the authors—the most straightforward way to limit the movements of empty containers. A street-turn means that the empty container does not pass the terminal but is immediately transported from the consignee to the consignor. The use of this strategy will reduce empty movements, and consequently, the repositioning costs will decline. Furthermore, the consignor’s demand for an empty container can be met sooner. This, in turn, will increase the container utilization rate. Legros et al. (2019) found that although it is inefficient, the detention charges and related activities such as cleaning incentivize consignees to return the container instantly to the maritime terminal operator. The detention fees act as a key barrier for street-turns as consignees believe that they do not have enough time to find an export match within the free detention period. This is in line with Storm’s (2011) finding that D&D limit the chance of re-utilizing a container. Another reason Legros et al. (2019) mentioned is that shipping lines are hesitant to allow strategies such as street-turns because they lose control over their equipment for a longer period, which might lead to missed opportunities.

##### Earlier announcement planning: information sharing

The container transportation chain is very complex, including many parties with different information flows. This leads to a lack of visibility regarding information and an information imbalance. Hence, to control D&D, information and documentation flows have to be shared among the different parties in the supply chain. If information is not shared, or only to a limited extent, D&D fees will be, in many cases, in danger of missing their target. Storm (2011), therefore, proposed transforming the process from reactive to proactive. For this, the various stakeholders involved in the process should share real-time data. Storm (2011) reveals that information sharing between the various partners can reduce lead and throughput times. The Federal Maritime Commission (2015) states, for example, that better preplanning and stowage on the vessel, vessel scheduling, regular vessel traffic information, and forecasts may contribute to the terminal operator’s capability to speed up the handling of import shipments. van der Welle (2015) looked at the development of a synchromodal planning tool for more efficient scheduling of hinterland transport, with an emphasis on the variables which affect D&D costs. The tool’s purpose is to show the shipper in advance the costs that can occur in the process, making it possible to plan the hinterland transport cost-efficiently. In this way, the shipper is not confronted with additional charges afterwards. Fazi and Roodbergen (2018) proposed integration of the ICT systems to improve the information flows and the use of combined D&D to increase flexibility, which will reduce the average dwell times.

##### Legislation

With respect to legislation, Schofield (2016) identified two major issues regarding the demurrage of the ship, which can also be applied to container D&D: 1) who is liable to pay the D&D fee and 2) which law from which jurisdiction should be applied.

It has become clear that terminal operators implement storage charges to reduce terminal congestion, and shipping lines implement D&D charges to keep the container equipment moving. But, who will pay these charges? Some disputes can arise: e.g., delays in the pick-up of containers due to inspections resulting in charges no party wants to pay; abandonment of the cargo by the consignee because the D&D expenses exceed the cost of the cargo, leaving the freight forwarder or shipper with the burden; customers unable or simply refusing to pay because they think it is not justified, etc. However, the broad definition of merchant in the Merchant Clause in the Bill of Lading gives the shipping line the right to impose D&D charges to a wide range of actors, i.e., shipper, consignee, truckers, forwarding agents, and notify parties as Merchant includes anyone acting on behalf of the shipper or receiver of the goods. Consequently, if one party refuses to pay, the shipping line can pass the charges on to another party, leading to conflicts (Schofield 2016; Magrath 2020; Munday 2021). As a result, the entire process of D&D is complex as the actor responsible for the demurrage and/or detention charges is often not the bearer of these charges (Chaudhri 2016).

In addition to the Merchant Clause, the Bill of Lading also contains a clause on which jurisdiction is applicable. As not all shipping lines operate under the same jurisdiction, disputes can be contradictory (Storm 2011).

In sum, the literature review of this research focused on three elements. First, a definition of D&D is given. It became clear that the terminology regarding D&D is often loosely used; therefore, Sect. "Results" describes a survey that probed for knowledge. The general idea behind D&D is quite clear; namely, the purpose of demurrage is to move the container as fast as possible through the terminal to maintain a certain level of terminal velocity. The purpose of detention is to achieve an efficient supply chain by facilitating equipment velocity. Second, the international commercial terms and the carriage of the container which are linked to D&D are discussed. Third, the organizational practices and challenges of terminals and carriers related to D&D are studied in-depth. The literature review shows that D&D is a globally used practice with different local applications. This research focuses on D&D experienced in Europe. Despite the straightforward goal of D&D, problems with and trends in D&D arise and are identified in various sector-relevant publications and, to a more limited extent, in the academic literature. Therefore, actions have to be taken. However, the solutions are often insufficient and are little to not problem-solving. To solve the issues related to D&D, more insight into the root cause is needed. For this reason, an industrial-economic perspective, including the interpretation of and experience in D&D by different actors in the maritime supply chain, is used. To conclude, The practice of D&D can meet its original purpose when every actor has the right and transparent knowledge and understanding, when it is correctly applied and when there is more standardization.

## Results

In this section, problem-focused thinking is replaced by solution-oriented thinking. In order to do so, the knowledge and the interpretation of the D&D challenges need to be identified. Therefore, a survey was distributed amongst the participants prior to the discussion meetings about the D&D issues.

In this section , first,  the profile of the different participants is presented, followed by the survey design. Then, the importance of elements that contribute to D&D are discussed as well as the ranking of the D&D cost elements. Laslty, Sect "Think about the solution: importance and feasibility" elaborates on the results of the discussion meetings and analyses the feasibility of the proposed solutions.

### Think about the solution: profile participants and survey design

D&D have an impact on several stages throughout the maritime supply chain. Following Storm (2011), this research adopts a supply chain approach.

A survey allows including the viewpoint of various actors in the maritime supply chain and comparing the knowledge and interpretation of the problems of D&D. Given the commercial sensitivity of the D&D matter, working with a survey allows gaining more in-depth insight rather than attributing blame to an actor. 53 surveys were sent to 11 target groups in Belgium. As participants of the same company were only admitted to the survey once, the response rate is 68%. The survey consisted of 17 questions (see Appendix 1). The profile of the respondents is freight forwarders (18%), shippers (16%), carriers (12%), logistics service providers (18%), associations (8%), others (IT, financial institution, port authority consultant, lawyer, city of Antwerp, university) (26%).

The first part of the survey (Q1–Q8) probes for the respondents' knowledge regarding and their practical experience with D&D. All respondents could broadly describe the separate terms demurrage and detention, and free time (Q1, Q2 & Q4). However, the combined export/import demurrage was somewhat unknown. More specifically, the answers range from being aware of D&D (shippers) to barely or unfamiliar with it (other actors) (Q3). Due to the loose use of terminology, respondents lack knowledge of the topic and are not fully aware of D&D implications. The problem becomes more acute for import containers. While meeting the closing is an unavoidable deadline for export containers (Q7). The topic of D&D is not always included in the tendering process (for instance, bounded by the terms and conditions of the freight forwarder). However, if problems arise (e.g., cargo blocked in a container due to customs control and/or other damage control) and the D&D costs quickly add up, the shipper will negotiate with the carrier about the order of magnitude of the total cost (Q5). In the case of a significant volume, the shipping companies are more lenient to waive the D&D cost. The size of volumes also plays a role in allowing extended free time by the shipping company at the shipper's request. Several factors can call for extra free time, i.e., lengthy customs formalities and deliveries by barge that take more time. Furthermore, a shift can be noticed from included storage and/or plug-in towards not included storage and plug-in in the demurrage charge (Q6). Whether storage and plug-in are included or not seems to depend on the carrier. Storage is an ‘out of pocket’ back-to-back cost recovery that the shipping company or shipping agent receives on behalf of the terminal. The terms ‘storage’ and ‘plug-in’ (eligible for temperature-controlled containers) are thus used in the contract between the shipping company and the terminal.

In the remainder of the survey (Q9–Q15), the interpretation of the D&D issue was questioned. Based on the literature review, firstly, the respondents were asked to rank according to importance the following seven elements: shortening of free time, increasing D&D fees, changed practices of terminals, terminal congestion, increasing ship size, lower (labor) productivity, and changed practices of shipping lines (Q9). The participants ranked the D&D problems from the highest (1) to the lowest (7). Table 1 gives an overview of the outcome for all actors except shipping lines and shipping lines separately. The ranking is divided into three intervals: the top (1–2), middle (3–4), and bottom interval (5–7) (see heading columns).

**Table 1:** Elements linked to D&D. *Source*: own compilation

Problem	Number of times ranked as (in %)					
	All actors (except shipping companies)			Shipping companies		
	1–2	3–5	6–7	1–2	3–4	5–7
Shortening of free time	47%	53%	0%	80%	20%	0%
Increasing D&D fees	42%	37%	21%	40%	60%	0%
Changed practices of terminals	24%	35%	41%	20%	60%	20%
Terminal congestion	37%	58%	5%	50%	50%	0%
Scale increase	26%	21%	53%	20%	0%	80%
Changed practices of shipping lines	33%	50%	17%	0%	60%	40%
lower (labor) productivity	6%	33%	61%	0%	40%	60%

Table 1 shows that the shortening of free time, the increase of the D&D fee, terminal congestion, and changed practices of shipping lines (at the headquarters level) are ranked highest. According to the respondents, these four elements have a vital impact on the D&D problem. Changed practices of terminals, increases in ship size, and lower (labor) productivity contribute, according to these respondents, less to the D&D problem. In the case of 'free time', the interpretation of the shipping companies does not differ from that of the other actors, while this is different in the case of 'scale increase'.

**Fig. 4:**
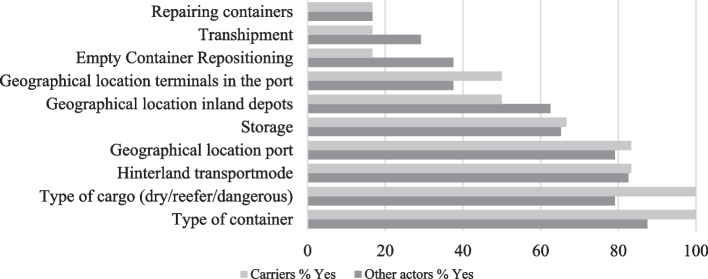
Cost elements (in %). *Source*: own composition

Next, the respondents (24) indicated which factors they believe form the basis for the D&D compensation (Q10). Figure 4 shows the importance of the cost elements for shipping lines and the other actors separately. According to all the respondents, the four main cost elements are the type of the container, the type of cargo (dry, reefer, dangerous), the availability of hinterland transport modes, and the geographical location of ports. According to the shipping lines, the two most significant cost drivers are the type of container and type of cargo. It is not surprising that the (type of the) container is seen as one of the most crucial (cost) elements forming D&D as the shipping line’s revenue model is based on the number of containers handled.

The shipping lines’ containers can be owned, which means that the shipping line made an investment cost or leased.^4^ In both cases, the cost for using the container during the entire transport is included in the shipping line’s freight rate (FIATA 2018). In the latter case, the shipping line will consider the lease cost per day that it has to pay to the leasing company. Hence, the leasing company's worry is not the commercial agreements regarding freight rate, free time, demurrage, and detention between the shipping line and the shipper. In general, it is all about capacity; and in particular, the late return of their container equipment. The late return of the shipping line’s owned containers can cause a shortage of capacity. In order to meet the customer’s demand, the shipping line will lease containers. However, when the leased container is returned late, the shipping line will have to pay the leasing company an extra fee—similar to the D&D principle—for the late return. D&D are thus compensations for the delay of the shipping line's owned containers and a provision for the costs that occur for the shipping line when a shipper returns a leased container too late. Hence, from the carrier's perspective, this approach (free time down/fee up) encourages that containers are being turned or speeding up the return of the container.

Then, the respondents are asked (Q11–Q15) about the impact of (merchant/carrier) carriage, Incoterms, whether D&D is hindering intermodal transport, who bears the costs/who are the benefits for, and which solution(s) might help to resolve the ongoing issues with D&D. Very different answers were given, rather indicating insufficient insight which needs further research; however in terms of solutions, there were many reactions. Each of the individual solutions is discussed in the following section.

### Think about the solution: importance and feasibility

Based on the literature review (a.o. Storm 2011; Chaudhri 2016), seven solutions that could help overcome D&D charges were formulated:

- Working days versus calendar days
- Increased negotiations
- Earlier announcement planning
- More free time for inland locations when using intermodal transport
- Digitalization
- Reuse of containers
- Legislation

The aforementioned solutions were proposed in the survey to the respondents. Analysis of the input shows that only a limited number of additional solutions were put forward, such as reliable and solid planning (system) to book slots for container collection, cheaper reuse, and uniformity in applying the selected solution.

To deliberate the survey results and assess the feasibility of each solution, actors from the maritime ecosystem of the Port of Antwerp were invited to a discussion moment. Fifty-three high-level managers responded to this call and took, spread over two sessions, a constructive part in the solution-oriented discussions. At the end of the discussion meeting, the participants were asked to rank the feasibility of the solutions. To do this, a range from zero (not feasible) to five (most likely to be feasible) was given. In addition, they were able to clarify the score they gave to each measure (see Appendix 2, columns 6 and 7) and which stakeholder they perceived was most likely to take the initiative or would be most involved (column 8).

**Fig. 5:**
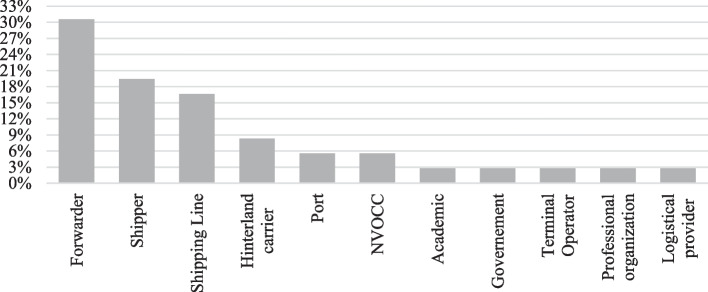
Amount of respondents per category feasibility ranking. *Source*: own composition

Figure 5 gives the number of respondents (36) per identified category who completed the feasibility ranking of the solutions. Together, they represent the different actors in the maritime supply chain. The discussions started from input from the survey. Since respondents from the same company were admitted to the feasibility ranking only once, the response rate was 68%.

Appendix 2 presents the responses regarding the perceived feasibility of the proposed solutions by all the actors. The discussion meetings show that the top three feasible solutions for D&D are: more free time for inland locations using intermodal transport, increased digitalization, and more attention during the negotiation process. Those are followed in order by reusing containers, using working days instead of calendar days, an earlier announcement of planning, and legislation. According to the feasibility ranking, the following sections explain both the results of each solution for D&D in more detail and the received reactions of the attendees.

#### More free time for inland terminals/intermodal transport

The first-ranked proposed solution provides more free time for inland locations when using intermodal transport. This measure was positively received by 30 of the 31 persons who shared their opinion on this question. The main reason cited in favor of this measure is that the use of intermodal transport is encouraged in this way. In addition, this ensures that hinterland transport can have a lower potential ecological footprint. Finally, this measure considers that the lead time when using rail or inland shipping is longer than when exclusively trucks are used. Therefore, the detention-free period should be extended, especially for merchant haulage. However, one needs to take into account the following three remarks to ensure that the feasibility of this solution is not negatively affected:

1. There is the dependence on the Incoterms2020, stating that for import, these are predominantly determined by the exporter.
2. It is necessary to have a sufficient number of empty depots in the hinterland.
3. Account must be taken of the fact that trade imbalances put a lot of pressure on the shipping lines to reposition their assets. Granting advantages for intermodal transport can make this optimization more complex.

#### Digitalization

The second-ranked solution concerns increasing digitalization in the sector. It is argued that there is a lack of information sharing between the different actors in the chain. Efficiency and accuracy can increase when data is shared in real-time and/or when a digitally shared planning tool is available for the different stakeholders concerned. This can transform the process from reactive to proactive.

A majority of the respondents estimated that the feasibility of digitalization is high (18 out of 34 indicated 5 on a scale of 5). Increased digitalization would enhance transparency and visibility. However, the respondents noted three major concerns. First, it was mentioned that a fragmented landscape of digital initiatives exists. Aligning those initiatives and making those systems uniform would be a challenge, especially when taking into account the fact that each company or organization would have invested a significant amount of resources into their projects. Second, the performance and security of the system are of key importance. Dependency on one system would be high, and the effects of a security breach such as hacking would mean that the chain would not be able to operate under normal circumstances temporarily, and all actors will be influenced. Third, many parties would be involved in the process of uniform systems. This would increase the complexity of decision-making.

#### Increased negotiations

The third-ranked proposed solution concerns increased negotiating. From the container shipping line perspective, this can be regarding specific large clients or trades, import or export, and mode of hinterland transport. The free period, as well as the D&D fees, can be the variables of the negotiation. 28 out of the 33 respondents indicated a feasibility score of three or higher on a scale of five for increased negotiation. The shipping lines and forwarders responded positively with six out of six and nine out of ten, respectively, indicating three or higher. This compared to the category shipper, where one out of seven responded with zero on a scale of five and one with a two on a scale of five. The reasons for the lower score are indicated in Appendix 2 and are the impression that there is no extra room for negotiating and that the way of negotiating has to be changed to be effective. Four out of ten forwarders mentioned that there was a key role for forwarders in the negotiating process.

#### Reuse containers

The fourth-ranked proposed solution is the reuse of containers. An example mentioned in this context is Avantida (acquired by E2open via INTTRA acquisition). The platform aims to lower the number of empty runs, i.e., transporting empty containers. An import container that is emptied at a customer is, in most cases, driven back to the port or an empty depot. Optimizations can be made by finding an export client nearby the import client and bringing the container directly to the export client. As a result, time, energy, and costs are saved. The majority of respondents (27 out of 34 indicated three or higher on a scale of five) had a positive view on this matter. The benefits are increased use of assets, less greenhouse gas emissions, and decreased congestion due to less transport. At the moment of the discussion, the costs to use the platform were perceived to be high. In addition, questions were raised regarding the process of checking the container for damages and the cleaning of containers destined for the transport of food.

#### Working days versus calendar days

The fifth-ranked solution consists of opting for working days instead of calendar days. The shipping lines worked with calendar days to determine the number of free time days and the D&D charges. It was argued that even though shippers and inland carriers were not fully able to collect or return the containers on weekends and public holidays, the shipping lines would charge D&D fees for these days. Therefore, this solution proposes to work with working days. This change would be from the perspective of the shipper and inland carrier and would increase the sentiment of fairness for those parties involved.

**Fig. 6:**
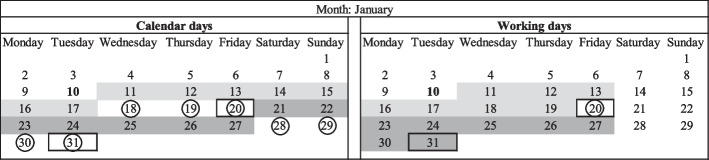
Example calendar—working days. *Source*: own composition

A practical explanation of this solution is given by the calendar shown in Fig. 6. Assume that the free time for demurrage is seven days, and the free time for detention is seven days. The container is released at the terminal on January 10th. On the one hand, the shipping line can apply calendar days to determine the D&D period. If the shipper or receiver does not want to be confronted with import demurrage, they will have to pick up the loaded container at the terminal on or before January 17th. Instead, the container is picked up on January 20th. This means import demurrage of three days. The detention period starts from the day the container is retrieved from the terminal, i.e., January 20th. Hence, the empty container must be back at the terminal on January 27th. Nevertheless, the container was only unloaded at the warehouse on January 28th due to a lack of space. As a result, the container is gate-in at the terminal on January 31st, and import detention is four days.

On the other hand, the shipping line can use working days to determine the D&D period. When the shipper or receiver does not want to be confronted with import demurrage charges, the container must be retrieved from the terminal on January 19th (weekend days are not counted). However, like in the previous case, the container is picked up on January 20th. This means import demurrage of one day instead of four days under calendar days. Similarly, the import detention period starts on January 20th. Consequently, with a seven-working day free period, the empty container must be back at the terminal on January 31st. The container was unloaded at the warehouse on January 28th due to a lack of space. As a result, the container is gate-in at the terminal on January 31st which means that import detention is zero.

The majority of the forwarders, shippers, and shipping lines estimate this measure’s feasibility to be positive. Ten out of eleven forwarders indicate a feasibility of three or higher. This ratio was six out of seven for the shipper’s category and five out of six for shipping lines. The terminal operator that decided to answer this question indicated a feasibility of three. However, the terminal operator later stated that this measure is not of interest to the terminal. This, in contrast, to manage the number of containers that are temporarily stored at the terminal, which is of high importance for this actor.

#### Earlier announcement planning

The solution ranked sixth consists of an earlier announcement of the respective planning. Respondents had a divided impression regarding the feasibility of this measure. The solution is related to digitalization and requires better communication between the various parties so that work can be done proactively. Proactive communication is essential for increasing the efficiency of the process of transporting containers in the hinterland. The possible identified obstacles to this proposed solution are external factors such as customs and the size and punctuality of the seagoing vessels.

#### Legislation

Possible changes in legislation are ranked last. The interest for this solution is lower as only half of the participants responded. Although 62.5% of the stakeholders (ten out of sixteen) estimated the feasibility to be positive (three or higher out of a scale of five), six out of sixteen respondents indicated a score of 2 or lower. The reasons in favour of more or changed legislation are an increase in clarity and the fact that uniformity of policies of the shipping lines can be accomplished. The reasons that withhold the success are that preferably it has to be determined at the European level and that customs have to be aligned in the process, which will take a significant amount of time.

### Who should take up a role?

Ultimately, the respondents were asked who should take on a role to convert the proposed solutions into actions. Their reactions are shown in the last column of Appendix 2. In order to implement these solutions, various actors have to work together:

1. Since D&D is a global problem determined at the headquarters level of shipping lines, possible changes should be discussed there, e.g., the shift from calendar days to working days to define free time.
2. More free time for inland locations when using intermodal transport and increased negotiations are accomplished in the relation carrier and shipper, or the actor acting on behalf of the shipper, e.g., the freight forwarder (associations). Particular attention should go to the negotiation process between these actors.
3. Increased digitalization can be achieved by the development of apps and the like that are made available through a data utility platform provider, e.g., NxtPort.

## Conclusions, recommendations, and further research

Due to recent events such as the bankruptcy of Hanjin, the cyber-attacks in the shipping industry, the COVID-19 pandemic (labor shortage, congestion, delays at ports and terminals, container availability), and the blockage of the Suez Canal, which resulted in a severe backlog of containers, the debate around Demurrage &Detention (D&D) came once again higher on the agenda as shippers’ agitation over increased D&D fees and shortening of free time is growing. In addition to these two factors, other factors may also contribute to the probability of D&D charges, namely increases in ship size, lower (labor) productivity, terminal congestion, and changed practices of terminal operators. The lack of uniformity and transparency of billing practices by terminal operators in their contractual relationship with shipping companies and, in turn, shipping companies in their contractual relationship with the principal of transport can confuse shippers about the imposed D&D charges.

The present paper clarifies commonly used terminology, describes factors that contribute to D&D, identifies the cost elements of D&D, and validates feasible solutions. Therefore, the research question arises whether D&D functions properly using a supply chain approach. Knowing that D&D is a globally used practice makes the matter more complex because decisions concerning D&D are made by the headquarters of shipping companies, but the impact is experienced at the local level. As the FMC and the EU have different scopes, the research chose to focus on local activities in European ports, more specifically, the port of Antwerp which is representative as second biggest port in Europe. When D&D is correctly applied and with the right, transparent and explicit knowledge, understanding, and standardization, D&D can meet its original purpose. However, to achieve this, several actions still have to occur.

The present research opts for a three-step solution-oriented approach for D&D issues. First, a literature review was conducted, which showed that only limited research on D&D exists. Furthermore, the literature review clarified that the definitions of D&D are loosely used. Subsequently, seven solutions emerged from the literature review and the semi-interviews conducted by Chaudhri (2016): (1) Working days versus calendar days, (2) Increased negotiations, (3) Earlier announcement planning, (4) More free time for inland locations when using intermodal transport, (5) Digitalization, (6) Reuse of containers and (7) Legislation.

The second step of the research included a survey and discussion meetings with high-level managers covering the different actors in the maritime supply chain. The survey consisted of 17 questions which can be divided into three large blocks: testing knowledge of commonly used terminology and discussing practical experience; interpretation of the D&D issue; and defining the cost elements of D&D. Firstly, the survey results confirm the literature review findings and show that the respondents insufficiently know the terms, especially the term combined demurrage. Next, according to all the actors, the D&D problem is mainly linked to shortening free time, increasing D&D fees, and terminal congestion. A clear distinction can be found in the answers of the shipping companies versus the other respondents (terminal operators, shippers, logistic service providers). Where the other actors link the changed practices of the shipping line (at the headquarters level) to D&D (83%), the shipping lines link it with the changed practices of terminal operators (80%).

Thirdly, analysis of the survey results provides insight into the cost elements of D&D or for what D&D compensates. According to all the actors, the three most significant elements are the type of container, type of cargo, and the availability of hinterland transport modes. The third step of the research also comprised the feasibility ranking of the solutions, including reasons in and out of favor during the discussion meetings. Out of the seven solutions discussed, the respondents ranked the following top three as viable: extra ‘free time’ for hinterland locations, digitalization, and more attention to possible D&D issues during the negotiation process. In the short run, those solutions can have an impact on the D&D fees and free period and, consequently, on the development of intermodal freight transport. In other words, the solutions can help with the modal choice/shift. To create more visibility in the maritime ecosystem and limit the impact of D&D practices on the development of intermodal freight transport, the different actors have to communicate, (re)negotiate, collaborate, coordinate, and co-innovate.

Ultimately, the research presented a detailed explanation and understanding of D&D, D&D-related difficulties confronting the actors in the maritime ecosystem, and solutions for the actors themselves and policymakers. The seven solutions provide relevant information and insights that can assist policymakers in taking action and putting the discussion about this issue on the right track. For policymakers, this is important because it creates awareness of the possible impact of D&D on intermodal freight transport, viz. avoiding reverse modal shift which in turn could lead to a failure of the applicable regulation. The research findings also contribute to discussing local experienced problems relating to D&D practices on a global level. In the context of the integration of the maritime chain, the outcomes of the research should actually be discussed by the industry stakeholders at the headquarters of the shipping companies and in close consultation with professional associations (shippers, freight forwarding, etc.). Next to policy and industry recommendations, for scholars, the present research gives more insight into the subject as only limited academic research on D&D is conducted.

Further research into D&D is needed to fully understand the impact on the maritime ecosystem as well as who bears the risks. Possible angles for further research are surveying on a larger scale, quantifying the feasibility of every solution, identifying in detail the cost elements of D&D, and exploring the operational issues for the sector due to COVID-19 and the blockage of the Suez Canal, which resulted in a severe backlog of containers. Another interesting path to consider is performing multidisciplinary research by including, to a greater extent, the legal impact, such as who is liable and what needs to be changed in the conditions. One more path is investigating whether the American law OSRA22 solves the D&D disputes and whether it should be aligned with the European approach. It could be useful for the sector to explore the problems of every actor in-depth. Could D&D be replaced by something else?

## Data Availability

The data from the interviews and discussion meetings used and analyzed during the current study are available from the corresponding author on reasonable request.

## Appendix 1: Research questionnaire

1. What do you understand under demurrage?
2. How do you define detention?
3. Are you familiar with combined D&D?
4. What do you understand by (extended) free time?
5. Do you negotiate demurrage, detention, combined D&D? If yes, please explain further.
6. Is storage, plug-in, … included or not?
7. Does import versus export have a varying effect on demurrage and detention?
8. How often are you confronted with D&D issues?
9. Academic research shows that the D&D problem is linked to the following elements. Rank these elements according to importance from 1 (main reason) to 7 (least important reason). Write your ranking in parentheses

**Table:**

a.	Shortening free time	(…)
b.	Increase of D&D fee	(…)
c.	Terminal congestion	(…)
d.	Lower (labor) productivity	(…)
e.	Increase in ship size	(…)
f.	Changed shipping company practices (at head office)	(…)
g.	Changed practices at the terminals	(…)
h.	*(additions are also possible)*	

10. Which factors form the basis for the compensation for demurrage and detention?* (strike through whichever is not applicable)*

**Table:**

a.	type cargo (dry/reefer/dangerous)	yes/no
b.	type container	yes/no
c.	geographic location	
	i. different ports,	yes/no
	ii. inland depots,	yes/no
	iii. terminals in a port	yes/no
d.	empty container repositioning,	yes/no
e.	transport modes hinterland transport	yes/no
f.	transshipment	yes/no
g.	container repair	yes/no
h.	storage	yes/no
i.	*(additions are possible)*	

11. What is the impact of the incoterms, carrier versus merchant haulage,…
12. Is the traditional use of D&D hindering multimodal/intermodal transport?
13. Which solutions (calendar days instead of weekdays, differentiate rates, more free time for containers to inland locations via intermodal transport, IT Technology (e.g., module within online platforms, use of sensors, immediately to warehouse (extra cost, with risk of shortage)? More transparency: how? Concept of subsystems, more correct freight rates,…) do you see the D&D problem and will this promote further integration of the maritime chain?
14. In the meantime, I was allowed to see some invoices, so that I could get an idea of the amount of the costs. Sharing your case studies will certainly contribute to academic research and discussion.
15. Who bears the costs? Who are the benefits for?
16. Are there any other things you would like to see discussed?
17. Would you like to present a problem briefly? (2 companies per session—10 min)

## Appendix 2: Overview solutions

**Table:**

Proposed measure/solution	Amount of respondents	Feasibility (scale from zero to five)			Reasons in favour of measure	Reasons out of favour of measure	Role?
		Average	Median	Percentage that indicated a score of three or higher			
Calendar days <> Working days	34	3.26	3	76.47%	Working days represent period when can be operated	Shifts the issue to the shipping line	Headquarters carriers
					Increase in transparency	Fear that free times will be reduced	
					Increase in simplicity	Only solution in the short run	
					Decrease pressure during weekend and public holidays	24/7 economy	
						Possible exception for Port of Antwerp?	
						Complex to implement uniform definition working day	
						IT systems must be adapted and work together	
Increased negotiation	33	3.79	4	84.85%	Need for clear agreements	No control when importing goods, exporting party negotiates terms	Carrier—Shipper (or acting on behalf)
					More attention is needed for the topic	Differences in negotiating power originating from size of the company and/or volumes	
					Opportunities regarding export flow	Depending on role in the chain, no possible negotiating power	
Earlier announcement of planning	28	3.21	3	75.00%	Increase efficiency	Customs	Maritime ecosystem (incl. customs)
						Size and punctuality of seagoing vessels	
More free time for inland locations via intermodal transport	31	4	4	96.77%	Encourages the use of intermodal transport	Dependant on incoterms	Carrier—Shipper (or acting on behalf)
					Intermodal transport has a longer lead time	Availability of sufficient amount of empty depots needed	
					Decrease environmental footprint	trade imbalances put significant pressure on shipping lines	
Increased digitization	34	4.09	5	88.24%	A higher degree of transparency	Fragmented landscape of current systems	Data utility platform (e.g. NxtPort)
					Increased visibility	Risks concerning hacking	
						Large amount of parties involved	
Re-use	34	3.76	4	79.41%	Increase use of assets	Current costs to use the platform are perceived to high	Association
					Less greenhouse gas emissions	Check for damages?	
					Fewer runs mean less congestion	How to re-use containers intended for the transport of food?	
Legislation	16	2.94	3	62.50%	Creating uniformity	EU level is desirable, takes time	Association
					Increased clarity	Customs have to be aligned, which takes time	

## Abbreviations

AMS: Automated Manifest System
a.o.: Amongst others
ATA: Actual time of arrival
CLECAT: European Association for Forwarding, Transport, Logistics, and Customs Services
CFR: Cost and Freight
COVID-19: Coronavirus disease
C/P: Charter party
D&D: Demurrage and detention
e.g.: Exempli gratia—For example
EU: European Union
ESC: European Shippers Council
ETA: Estimated time of arrival
Etc.: Et cetera—and the rest
ETS: Estimated time of sailing
EXW: Ex Works
FIATA: International Federation of Freight Forwarders
FMC: Federal Maritime Commission
i.e.: Id est—that is
Incl.: Including
IT: Information technology
ICT: Information communications technology
Incoterms: International Commercial Terms
MTO: Maritime terminal operator
NVOCC: Non-Vessel-Operating Common Carrier
Q: Question
SCFI: Shanghai Containerized Freight Index.
TEU: Twenty-foot equivalent unit
US: United States of America
VGM: Verified Gross Mass
VOCCs: Vessel-operating common carriers
Vs.: Versus

## Footnotes

1 In the last century, the shipping companies offered their customers a free time usually between 14 and 21 days. Because port/terminal capacity became scarcer, the number of days was reduced to typically 7 to 10 days. In the last decade, some shipping companies have reduced this to 3–5 days.

2 On 22 April 2022, the port of Antwerp and the port of Zeebrugge merged (Port of Antwerp-Bruges 2022). The former port of Antwerp is used for the survey and discussion meetings.

3 OSRA22’s goal is to ensure a (cost)effective, competitive, and fair maritime transportation system by increasing the visibility of maritime shipping industry practices, shipping line requirements, and government power. OSRA22 has given the FMC specific timelines and increased authority to implement the new law provisions and initiate D&D rulemaking. An example of a rule to increase the visibility of D&D charging practice which was effective immediately is the inclusion of 13 provisions in the D&D invoice: the date that the container is made available, the port of discharge, the container number or numbers, for exported shipments, the earliest return date, the allowed free time in days, the start date of free time, the end date of free time, the applicable detention or demurrage rule on which the daily rate is based, the applicable rate or rates per the applicable rule, the total amount due, the email, telephone number, or other appropriate contact information for questions or requests for mitigation of fees, a statement that the charges are consistent with any of Federal Maritime Commission rules concerning detention and demurrage, a statement that the common carrier’s performance did not cause or contribute to the underlying invoiced charges. However, some aspects of OSRA22 still need to be clarified. While OSRA22 provides remedies for specific issues, it cannot address all supply chain disruptions e.g. the operational inefficiencies of US ports which also affect D&D (Senate—Commerce, Science, and Transportation, 2022).

4 Based on Drewry and Containerisation International data (2017), newbuild dry cargo container prices (China/ex factory) fluctuates around an average of 2,000 USD/TEU for 20ft standard containers and almost double this price for a 40ft container over the period 1999–2019; while a reefer container quickly costs + 12,000 USD per TEU. Container lease rates vary, depending on supply and demand, between average per diem rental rates of 0.4 USD/day and 0.8 USD/day (Drewry’s Container Census and Leasing Industry Annual Report, 2017). The trade-off can easily be calculated taken the number of days at port of origin + transit time + number of days at port of destination (for instance: 14d. at port of origin, 35d. transit time, 14d. port of destination or 63d.).
